# Assumed shared belief about conspiracy theories in social networks protects paranoid individuals against distress

**DOI:** 10.1038/s41598-023-33305-w

**Published:** 2023-04-13

**Authors:** Praveen Suthaharan, Philip R. Corlett

**Affiliations:** 1 Interdepartmental Neuroscience Program, Yale University, New Haven, CT USA; 2 Kavli Institute for Neuroscience, Yale University, New Haven, CT USA; 3 Department of Psychiatry, Connecticut Mental Health Center, Yale University, New Haven, CT USA; 4 Department of Psychology, Yale University, New Haven, CT USA; 5 Wu Tsai Institute, Yale University, New Haven, CT USA

**Keywords:** Human behaviour, Risk factors

## Abstract

Paranoia is the belief that others intend you harm. It is related to conspiracy theories, wherein those others represent an organized faction, coordinating the harm against self and others, and violating societal norms. Current psychological studies of paranoid conspiracy theorizing focus either on the individual or their broader social network. Likewise, theories of belief formation and updating often contain individual level processes as well as broader interpersonal and organizational factors. Here we examine paranoia and conspiracy theorizing in terms of individual behavioral predictors (performance on a probabilistic reversal learning task which assays belief updating) as well as social sensing: we ask participants to report the features of their social network, including whether their friends and acquaintances share their paranoid conspiratorial beliefs. We find that people who believe paranoid conspiracy theories expect more volatility during the task. They also assume that members of their social network share their paranoid beliefs. Critically, those participants with larger social networks and greater assumed shared belief tend to harbor their conspiratorial beliefs with less emotional distress and expect less volatility in the task. This is evidence that, like political and religious beliefs, conspiracy theories may flourish under a sacred canopy of belief consensus. These data suggest that friends and acquaintances may serve as sources of credulity and moving between them may sustain conspiracy beliefs when there is detraction. This hybrid individual/social account may shed light on clinical paranoia and persecutory delusion, wherein disability is defined normatively, and social supports are fewer.

## Introduction

Paranoia—the belief that others intend to harm us, and conspiracy theories; beliefs that powerful groups are colluding against our interests, are related^1^. Both have flourished during the uncertainty and social upheaval we have experienced during the past two years of the COVID-19 pandemic^2^. Both types of belief also seem resistant to change in the face of overwhelming contradictory evidence. Whilst some people find these beliefs extremely distressing, others do not. Understanding this distinction may be key to discerning extremities of unusual belief from the delusions that occur in psychotic illness^3^. We sought such understanding by focusing on the relationships between individual cognition and paranoia, and the assumptions made about others’ beliefs in conspiracies.

Contemporary accounts of belief formation and updating posit individual-level and social-network factors which govern whether a particular belief is adopted and maintained^4^. For example, paranoid people^5,6^ and those who endorse COVID-19 vaccine conspiracies^2^ expect more volatility in the world and update their beliefs erratically. Furthermore, conspiracy theories spread rapidly through social networks^7^. However, individual differences in psychological processes (like prior beliefs about volatility) are rarely examined simultaneously with the features of those individuals’ social networks (see^8,9^ for notable exceptions, however neither study examined belief updating). This is a pressing issue, given the societal consequences of paranoid beliefs and conspiracy theories^10^. We measured paranoia, conspiracy theorizing, task-based belief updating, and perceived social network properties in the same participants.

Such data are of theoretical import. There are accounts of paranoia that implicate social-specific mechanisms (like coalitional threat)^11^. There are other theories that posit domain-general mechanisms^12^. Domain-general theories are often criticized because the link from general learning mechanisms to a specific social concern is harder to draw. However, the data favoring social-specific processes over general ones are weaker if not absent^13^. Establishing the relationship between general mechanisms, social-specific processes, and experiences of social relationships, will help clarify what underwrites paranoia and conspiracy theorizing. Moreover, neither account has explained why paranoia and conspiracy theories are so resistant to change in the face of contradictory evidence and dissenting opinions. This is not simply an arcane academic exercise. Armed with a deeper understanding of the mechanisms underlying paranoid conspiracies, we will be better placed to intervene—either at the level of individual adherents, or through their social networks. Successfully mollifying conspiracies will mitigate distress and suffering^10^.

Modern social science has recognized the importance of human social-sensors^14^; individuals who are asked to report their subjective beliefs about their immediate social environments. Sociologists do not typically ask people what they believe their social contacts believe, perhaps because individuals who support a particular view often inflate their estimates of the view’s prevalence, relative to non-supporters^15^. However, such false consensus beliefs may explain why conspiracy beliefs persist. Furthermore, egocentric social sensor data regarding the perceived flu vaccination behavior of social contacts predicted the sensor’s own vaccination likelihood a year later. It would seem critical to examine whether the same might hold for COVID vaccine beliefs. People who believe that the COVID vaccine has many unpleasant side effects also report perceiving less of a social norm for vaccination^16^. Social sensing research may enlighten the coupling from personal beliefs and behaviors to norms, with direct public health consequences.

Paranoid people tend to assume that others like them will also endorse conspiratorial beliefs^17^. However, we do not know how those assumptions relate to their specific social networks. Furthermore, empirical and computational work has shown that participants’ perceived similarity between themselves and the partner with whom they are interacting mechanistically influences social interaction by generating more accurate predictions and less threatening impressions of the partner^18^. Paranoia rendered such alignment more challenging^18^. This disconnect emphasizes the importance of relating task-elicited behaviors to real-world social understanding if we are to explain paranoia and conspiracy theories more completely.

Endorsing conspiracy theories is associated with a greater social network diversity and smaller social network size^19^, however, there are more comprehensive methods for characterizing what individuals believe about their own social network structure, and the beliefs of the people with whom they interact. We modified an online survey tool (inspired by work in multiple sclerosis^20^), to ascertain individuals’ social networks and their beliefs about what members of the network believe. First, participants (termed ***egos*** in this literature) named all the people with whom they have discussed important matters, socialized, or sought support in the last 3 months (their ***alters***). Next, they answered questions to evaluate the connections between each pair of the first ten persons in the network, including the strength of ties between them. Finally, they answered questions about the beliefs of each of the first ten persons in the network (do the egos believe that the alters share beliefs about conspiracies? We sought to determine whether those beliefs differed by the paranoia level of the ego).

**Figure 1:**
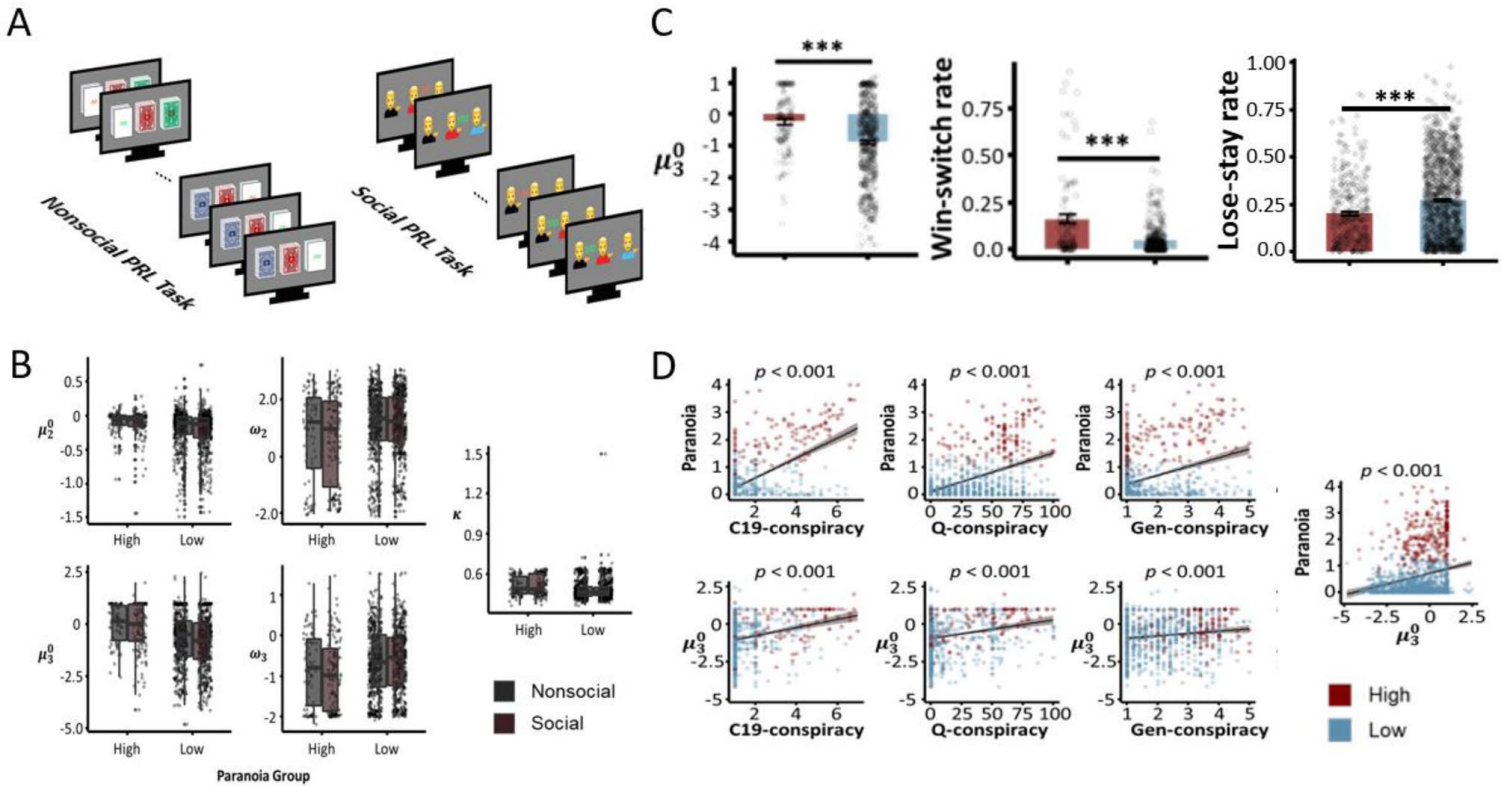
Probabilistic reversal learning behavior, beliefs, and paranoia. We replicate the relationships between task performance, paranoia and conspiracy beliefs. (**A**) *Probabilistic reversal learning task.* Behavioral paradigm used to measure belief-updating under uncertainty. Participants completed a non-social (card decks, n = 831) or social (collaborators, n = 707) version of the probabilistic reversal learning task (**B**) *Between-task replication*. As shown previously^2^, we see no effect of task frame (social vs nonsocial) on beliefs. (**C**) *Paranoia group differences in task behavior and belief.* Win-switching behavior, lose-stay behavior and task belief volatility differ between high and low paranoia individuals. Paranoid individuals expect more volatility going into the task ($W=275568, p<0.001$), more frequently switch to different decks even after winning ($W=284409, p<0.001$), and less frequently stay on the same deck even after a loss ($W=161204, p<0.001$). (**D**) *Relationships between conspiracy theorizing, paranoia, and task derived volatility beliefs.* We replicate the relationship between covid-19 vaccine conspiracy belief and paranoia ($\uprho =0.42,p<0.001$; top-left), QAnon conspiracy belief and paranoia ($\uprho =0.30, p<0.001$; top-mid), general conspiracy belief and paranoia ($\uprho =0.41,p<0.001$; top-left), covid-19 vaccine conspiracy belief and ${\mu }_{3}^{0}$ ($\uprho =0.29,p<0.001$; bottom-left), QAnon conspiracy belief and ${\mu }_{3}^{0}$ ($\uprho =0.21, p<0.001$; bottom-mid), general conspiracy belief and ${\mu }_{3}^{0}$ ($\uprho =0.17, p<0.001$; bottom-left), and paranoia and ${\mu }_{3}^{0}$ ($\uprho =0.24,p<0.001$; right).

We also assessed participants belief updating using a non-social and social probabilistic reversal learning task (Fig. 1A), performance on which we have previously related to paranoia and endorsement of COVID-19 vaccine conspiracy theories^2^. Participants chose between 3 decks of cards (or avatars) with different reward probabilities to learn the best deck (or avatar)^21^. Participants were forewarned that the best deck (or avatar) may change, but not when or how often^21^. Furthermore, part way through the task, the underlying probabilities became more difficult to distinguish, increasing unexpected uncertainty and blurring the distinction between probabilistic errors and contingency shifts. The challenge is to harbour beliefs that are robust to noise but sensitive to real contingency changes^21^ (See “Methods” for more detail).

Probabilistic reversal learning involves decision-making under uncertainty—uncertainty about which option to choose and whether the options have recently changed or even reversed in their value. By studying people’s choices with computational modeling, we can try to understand the latent processes (or beliefs) that led to those choices. We think that people form and update a number of beliefs about the properties of the task, which help guide their choices. The computational model we fit to participants’ choices, and whose parameters we estimate, has two components: participants’ assumptions about how the task works comprise one, the other governs those assumptions lead to their decisions. The model is named a Hierarchical Gaussian Filter (HGF). In this model the component that incorporates task beliefs is called the **perceptual model**. The component that governs how beliefs are converted into choices is called the **response model**.

The perceptual model has three hierarchical layers of belief about the task. The layers interact and influence one another through learning rate parameters. At the highest level (i.e., level 3), the model captures beliefs about changes in the task environment (how are values of the choices changing over time?). Level 2 characterizes beliefs on reward probabilities (i.e., the tendency of a choice to be rewarding). Level 1 characterizes task reward feedback (i.e., win or loss). These three levels of belief are then integrated and fed through a sigmoid response function to produce a decision (whether to either stay with the same card deck (or partner) or switch to a different one).

We previously showed^2^ that there were no model parameter differences between the social (avatar) and non-social (card deck) versions of the task and their relationship to paranoia. We first set out to replicate these findings. In a subsequent study, we focused on a single parameter that we have found, across multiple studies, to be a key marker of paranoia—the initial prior belief on task volatility (${\mu }_{3}^{0}$). Participants with larger values of this parameter are entering the task anticipating more changes in the task environment (e.g., expecting rewarding decks to change—reversal events). In this paper, we used this task and the individuals’ estimated prior belief about volatility to explore the relationships between belief-updating, conspiratorial beliefs and social network features.

These data allow us to test key hypotheses about social network properties and conspiracy beliefs. For instance, political and religious beliefs^22^ appear to be protected by a ‘*sacred canopy’*^23^; a socially constructed plausibility structure of close individuals who we assume share our convictions. This canopy protects those convictions from dissonant information. Having social connections to people who share one’s ideology increases the strength of shared beliefs^24^. We aimed to establish whether paranoid conspiracies were also protected by a sacred canopy of assumed shared belief.

Furthermore, the nature of the connections may be critical to how beliefs are shared through social network^25^. Weak ties are thought to facilitate the diffusion of information through networks because of their tendency to span otherwise distant subgroups. However, due to a lack of trust, an unwillingness to share benefits, or a limited ability to understand one another, an individual is less likely to share novel information across weak ties^25^. As networks become more structurally diverse (accumulating weak bridging ties and forgoing strong cohesive ties), the bandwidth of their communication should contract, reducing information flow. This may protect misbeliefs against dissonant information.

We aimed to establish (1) the degree to which paranoid individuals believe that others share their conspiratorial beliefs, (2) whether paranoid people report social networks with particular types and densities of ties, and (3) whether individual belief updating metrics relate to paranoia, conspiracy beliefs, sacred canopies and other social network features.

## Results

We replicated the previously published^2^ relationships between task performance, paranoia and conspiracy beliefs. Again, we found no effect of social (partners) vs non-social (card decks) task frame on the associations between behavior and self-reported paranoia (Fig. 1B, paranoia was measured with a standard self-report scale, and high paranoia was defined according to a cutoff that would be of clinical concern^26^). However, more paranoid individuals expected more volatility going into the task (${\mu }_{3}^{0}; W=275568, p<0.001$), more frequently switched to different decks even after winning (win-switch rate; $W=284409, p<0.001$), and less frequently stayed on the same deck even after a loss (lose-stay rate; $W=161204, p<0.001$) (Fig. 1C). We replicated the relationship between covid-19 vaccine conspiracy belief and paranoia ($\uprho =0.42,p<0.001$), QAnon conspiracy belief and paranoia ($\uprho =0.30, p<0.001),$ general conspiracy belief and paranoia ($\uprho =0.41,p<0.001$), COVID-19 vaccine conspiracy belief and ${\mu }_{3}^{0}$ ($\uprho =0.29,p<0.001$), QAnon conspiracy belief and ${\mu }_{3}^{0}$ ($\uprho =0.21, p<0.001$), general conspiracy belief and ${\mu }_{3}^{0}$ ($\uprho =0.17, p<0.001$), and paranoia and ${\mu }_{3}^{0}$ ($\uprho =0.24,p<0.001$) (Fig. 1D).

**Figure 2:**
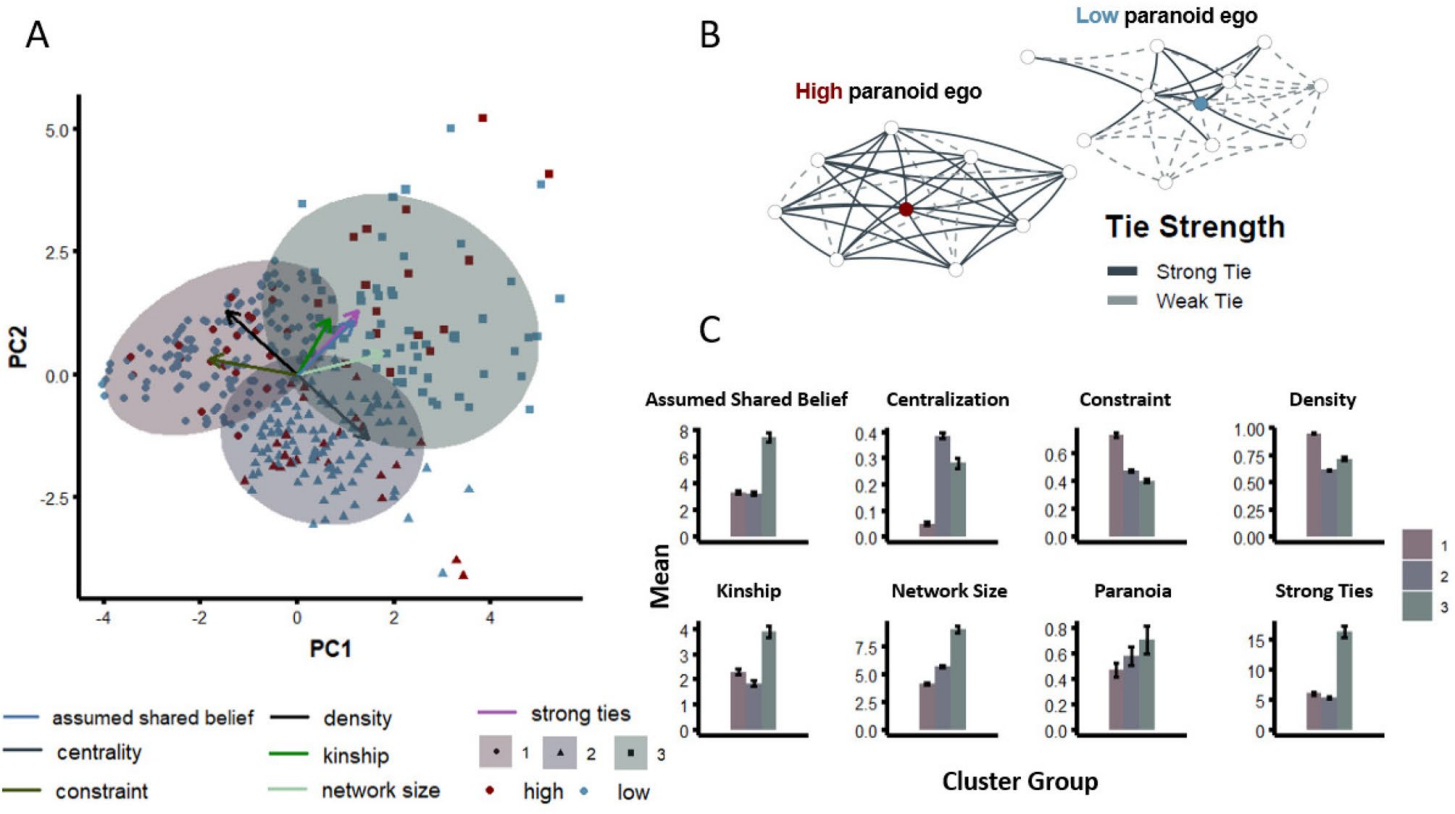
Beliefs about social relationships and shared beliefs relate to self-reported paranoia. (**A**) *Principal Components Analysis Bi-plot*. We performed k-means clustering ($k=3$ optimal clusters, based on the Elbow test) on 7 different network features (network size, strong ties, centralization, constraint, density, kinship, and assumed shared belief). We visualize the first two principal components as dimensions. The *direction* of the vector loadings of each feature illustrate which feature drives which cluster. For example, assumed shared belief, strong ties, network size, and kinship distinguishes cluster 3 from the other clusters. (**B**) *High paranoia and Low paranoia networks*. For illustrative purposes we depict a typical network described by a high paranoia participant and a low paranoia participant. Paranoia is characterized by more and stronger self-reported ties. (**C**) *Distribution of features across clusters*. We note that cluster 3 has the strongest assumed shared belief, kinship, network size, paranoia, and strong ties.

Having solicited participants’ beliefs about their social networks (including whether they believed alters in their network shared their beliefs about various conspiracies), we performed k-means clustering ($k=3$ optimal clusters, based on the Elbow test; see “Methods” for more detail) on 7 different network features (network size, strong ties, centralization, constraint, density, kinship, and assumed shared belief; see “Methods” for more detail) (Fig. 2A). Having a larger network and greater kinship distinguished cluster 3 from the others. Figure 2B depicts characteristic networks from Egos with low and high paranoia. Note the greater number of reported weak and strong ties. Cluster 3 was also characterized by the strongest assumed shared belief, strong ties and paranoia (Fig. 2C).

When we mean split participants into those who assumed people in their social network shared their beliefs and those who did not, we find little to no differences in task beliefs ($B{F}_{10}=0.163$; moderate evidence for the null hypothesis that there was no difference in task beliefs between those with high versus low assumed shared belief) or combined psychopathology (depression, anxiety, and paranoia, $B{F}_{10}=1.37$; anecdotal evidence of no difference in psychopathology).

A key aspect of the sacred canopy theory is that the protective benefits are mediated through close social ties. To examine for a sacred canopy of assumed shared belief, we therefore employed multiple regressions to explore the more complex interactions between assumed shared belief, self-reported social network features and task beliefs.

**Figure 3:**
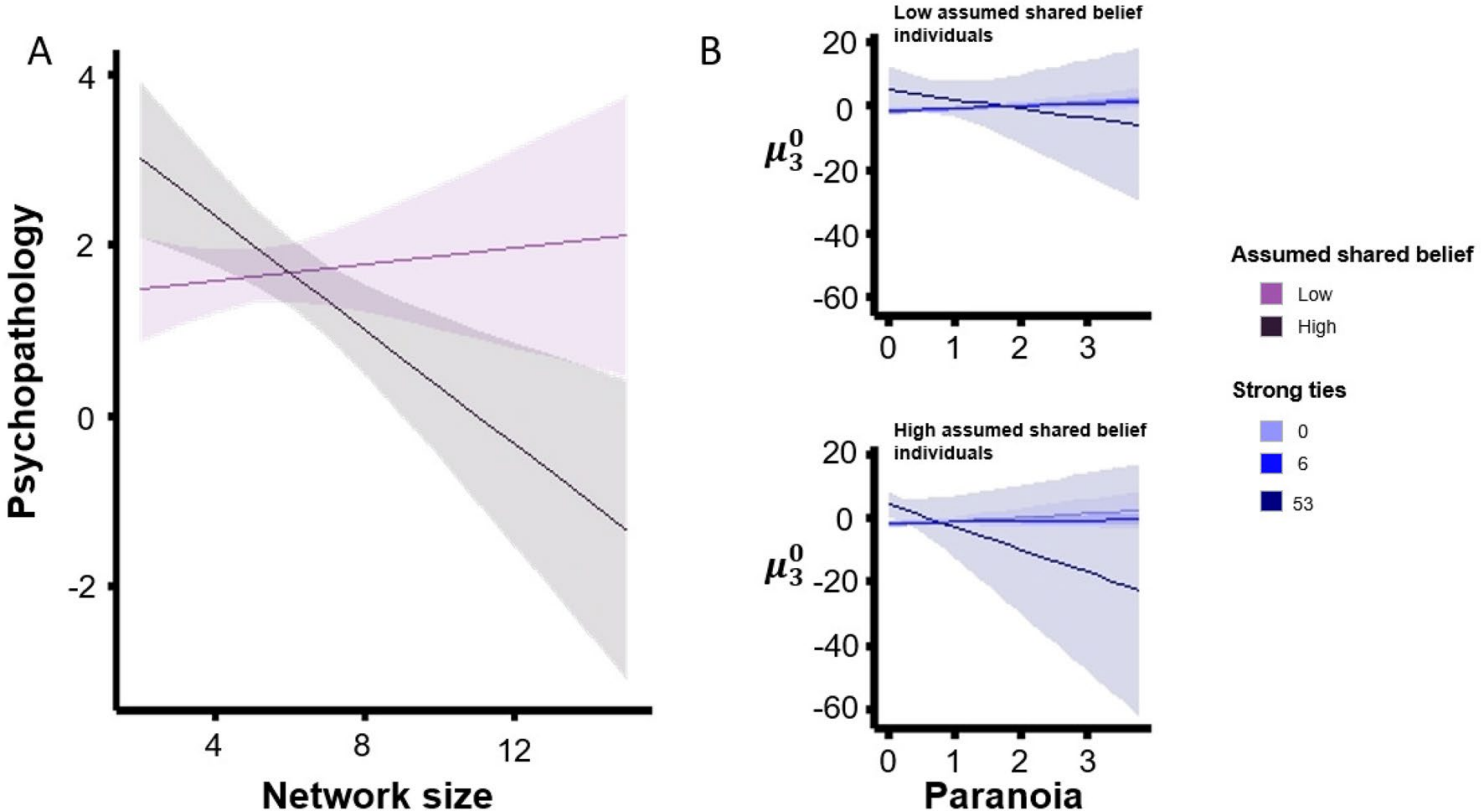
Protective effects of social support on psychopathology. Individuals with larger social networks, more strong ties and greater assumed shared belief show less psychopathology and expect less task volatility. (**A**) *Two-way interaction*. When assumed shared belief is high, psychopathology decreases with network size. When assumed shared belief is low, that is not the case (${F}_{(\text{7,195})}=4.03, p= 0.01$) (**B**) *Three-way interaction*. The relationship between paranoia and volatility belief depends on the perceived strength of ties and assumptions about shared beliefs. When assumed belief is high, the relationship between paranoia and volatility belief is negative—suggesting that more paranoid people embedded in what they perceive to be a sacred canopy switch their task beliefs less erratically (${F}_{(\text{13,189})}=3.97, p= 0.03$). Note: assumed shared belief grouping is based on a mean split, and strong ties are grouped based on min, median and max of number of strong ties.

Individuals with larger social networks, more strong ties and greater assumed shared belief reported less psychopathology (i.e., less anxiety, depression and paranoia), and expected less task volatility. When assumed shared belief was high, psychopathology decreased with network size (Fig. 3A). When assumed shared belief was low, that was not the case (${F}_{(\text{7,195})}=4.03, p= 0.01$). Furthermore, the relationship between paranoia and volatility belief depended on the perceived strength of ties and assumptions about shared beliefs (Fig. 3B). When assumed belief was high, the relationship between paranoia and volatility belief was negative—suggesting that more paranoid people embedded in what they perceive to be a sacred canopy expected less volatility in the task (${F}_{(\text{13,189})}=3.97, p= 0.03$).

Political beliefs may also anchor a sacred canopy^27^. As a check for the propriety of our methods, we sought to replicate these effects. Republican egos assumed that more members of their social network shared their beliefs about the right-wing QAnon conspiracy (Supplementary Fig. 1). Republicans with greater assumed shared belief also experienced numerically less psychopathology, however the difference was not significant. There may be at least two explanations for this: (1) our sample size of republicans was small, and (2) the effects of assumed shared political belief may be moderated by the local political climate (a canopy may be more important, but perhaps less effective if one lives in a state where ones’ favored party is not in power). We previously found such effects with regards to Pandemic policies^2^. Future work with larger geographically stratified samples will pursue these ideas.

Self-reference—the extent to which one is the focus of the conspiracy, is one feature that some posit is more relevant to clinical delusions than paranoia on a broader continuum^28^. However, in our data people with greater referentiality were no differently protected by the sacred canopy of assumed shared belief (Supplementary Fig. 2), that is, more delusion-like beliefs were not more common in individuals with more sparse social networks or weaker canopies of shared belief. This bears investigation and replication in people with clinical delusions.

## Discussion

We replicate and extend our previous work connecting prior beliefs about volatility (µ^0^_3_) in a behavioral task to concerns about other people (paranoia and conspiracy theories). These laboratory data again related to participants beliefs about the real-world. People who were more paranoid reported social networks with high degree centrality. This means that they described more social connections to themselves. They reported more weak ties to themselves and in their networks. They also reported more strong ties. This is perhaps surprising. First, the popular preconception is that paranoia and conspiracy theorizing is associated with loneliness and isolation^28^. This does not seem to be the case in our data. Of course, it is possible that people with paranoia assume that they have more friends and acquaintances than they really do. Our method cannot speak to the veracity of participants’ beliefs about their networks. Nevertheless, assuming more interconnectivity between oneself and others was a feature of more paranoid participants.

More paranoid people endorsed more conspiracies themselves. They also assumed more members of their social network shared their conspiratorial beliefs. Intriguingly, people who assumed more shared belief with others had more erratic task behavior and stronger volatility priors. However, the relationship between paranoia, erratic behavior, and expected volatility was moderated by reported social network size. People with larger social networks and more assumed shared belief had weaker prior beliefs about volatility in the task. Furthermore, those people with more assumed belief and bigger reported networks also reported less depression, anxiety and paranoia. This suggests that—like religious and political beliefs—paranoid conspiracy theories function under a sacred canopy^22,23^; an assumed shared belief structure that conveys support, emotional benefits, and protection against dissonance and disagreement. This aligns with broader qualitative work in the social sciences suggesting that conspiracy theories (like QAnon) have become more like religious beliefs^29^.

How might volatility beliefs and social network properties interact in this way to protect against negative emotions and dissonance? People who harbor anti-vaccine beliefs, for example, do not update their stance on vaccines when confronted with evidence that vaccines do not cause autism. Instead, their anti-vaxx stance apparently backfires, leaning more heavily on other accessory hypotheses associated with their anti-vaxx beliefs (like vaccines contain toxic heavy metals and so remain bad, without causing autism)^30^. Having lots of accessory hypotheses is a useful approach to never being wrong^31^. Whilst backfire effects may not be the route to maintaining wrong beliefs, the impact of different social sources of support for one’s beliefs is still relevant^32^. The present data suggest other people might serve as accessory hypotheses, alternative sources of support for a belief, to which one may turn when others friends disavow the belief. If someone disagrees with you, it is helpful to believe you have a large stable of other friends and acquaintances with whom you still agree. Perceiving a large network of like-minded social connections appears to obviate the need to switch allegiances more erratically and was associated with having weaker priors on volatility in the behavioral task. This is an admittedly reductionist view of social influences on belief. We do not deny that more complex possibilities (including theory of mind-based simulation and strategic signaling) are possible and important. But our data continue to suggest that paranoia and conspiracy theorizing—even the social aspects thereof—are related to low-level domain-general primary learning mechanisms^33^ which may be dopaminergically and noradrenergically mediated^34,35^. This domain general contribution to paranoia involves learning expectancies about the environment and the agents within it^36^, a process that may be guided by using oneself as a source of prior beliefs^18^. Our data represent an extension of those processes into real world social relationships.

Our data also align with ethnic density effects on psychosis risk and paranoia in particular^37^. People who identify as ethnic minorities are particularly prone to psychosis, especially when they are surrounded by fewer people who share their ethnicity. Our data suggest that shared beliefs may function like shared ethnicity; offering protection when they are dense, by way of community support, and increasing paranoia and distress when they are sparser. Taken together with our prior work on the effects of the evolving pandemic, we believe we have identified a solution to an apparent paradox: people who endorse conspiracy theories tend to feel that there has been a decline in society, especially with regards to rule-following which makes them distressed. Our prior work suggested that erratic behavior and paranoia flourished in the face of rule breaking (failure to follow mask-wearing guidance in states with a mask mandate where rule following was valued). The present work suggests that assuming that those around you think like you protects against erratic behavior. Perhaps this sacred canopy of assumed shared belief allows the invention of idiosyncratic rules, which protect against the deleterious effects of paranoia and distress. We note with interest that patients with schizophrenia tend to have smaller, sparser social networks and by definition less assumed shared belief (since delusions are not shared by same culture peers). Our present work suggests one mechanism through which peer support of people with serious mental illness by people with serious mental illness may be effective.

## Limitations

Our social network measures are all derived from egos, expressing their memories of, attitudes towards, and beliefs about their self-reported alters. We were not able to confirm whether they were actually alters. Furthermore, social networks carry with them an implication of dynamics—how, for example, beliefs pass between nodes and along edges. This is not our present concern. This is a static snapshot, from the ego perspective, on what they believe to be their social network, and the beliefs of their alters. To build outwards from ego properties to network properties, we felt it important to start here. In future work we will ascertain whether the alters do indeed share the ego’s beliefs. Any disconnect between egos’ assumptions and alters’ reports will be critical to intervention efforts. Disagreements with people with whom we share strong social bonds and who we assumed thought like us may provide the strongest impetus to belief updating.

Furthermore, these are correlative data. We cannot make claims about causal direction. To do so would require time-series data. People might surround themselves with like-minded people (homophily)^38^. Following participants over time and capturing the connections that form and break, as well as the behavioral and psychopathological consequences of those changes, will be key to understanding how individual cognition shapes beliefs about friends and acquaintances and vice versa.

Paranoia and conspiracies are not necessarily synonymous and may have unique predictors^19^. Nevertheless, they are correlated and here, as before, we have explored their shared variance. Our data suggest that harboring conspiracy theories is not always distressing, if they are harbored in the context of a richer set of social connections with whom one can assume consensus. Finally, delusion may not merely be an extreme form of conspiracy theorizing. It appears that delusions might implicate the sufferer more directly in the plot^1,28^. However, our effects were carried by the persecutory aspects of paranoia rather than by the extent to which events refer to the self (Supplementary Fig. 2). Nevertheless, by replicating this approach in patients with persecutory delusions we will establish the relevance of these findings for psychosis more broadly and the importance of self-reference in delusional beliefs.

## Conclusions

Paranoid conspiracy theories can be maintained without distress if one assumes that friends and acquaintances share the beliefs. Like political and religious beliefs, conspiracies seem to be protected by a sacred canopy of shared belief which neutralizes the depression and anxiety which commonly attends believing lots of conspiracy theories. The canopy also seemed to mitigate the relationship between paranoia and laboratory task behavior and model parameters. People with larger social networks and more assumed shared conspiracy belief expected less volatility during a probabilistic reversal learning task.

These data may have implications for attempts to change people’s minds about conspiracy theories. It may be most impactful to deliver a message of disagreement from someone who is a strong tie that the ego believes shares their belief. This would induce the largest prediction error. However, whether or not that error is accommodated or assimilated will depend on its precision. Therefore, picking multiple people that the ego assumes share their conspiracy belief—rather than just one—may ultimately prove most effective.

More broadly, these social network data may have promise for distinguishing conspiracy theories from clinical delusions. Meta-analysis of social networks from patients with schizophrenia suggest they have fewer network connections^39^. Typically, persecutory delusions are defined as beliefs about the intentions of others that are not shared by same culture peers. Studies of new religious movements have identified individuals who are as convinced and preoccupied by their bizarre beliefs as people with clinical delusions, however, they are rarely as distressed as these clinical cases^40^. Considering our present data, this may be because they are embedded in a network of likeminded individuals who concur with their unusual belief, whereas patients are not. We recently showed that people with paranoid delusions (rather than conspiracists) have strong prior beliefs on volatility^41^. They may also have smaller social networks and less assumed shared belief in paranoid conspiratorial ideas—manifest ultimately as a distressing state. At the onset of psychotic illness, social networks may contract, with connections being lost^42,43^. It would be interesting to characterize these social network and belief updating metrics as predictors of persecutory delusion in the clinical high risk prodrome^44^. The rare syndrome of Folie a Deux—or shared delusion—may be another informative example. Here, a non-psychotic proband (relative, spouse, or friend) comes to share the delusion of someone with psychosis^45^. It typically occurs in the context of a close social relationship and social isolation. Both proband and patient experience significant distress. While our data do not yet distinguish delusions from other conspiratorial beliefs, they do suggest querying individuals about their social networks may be informative with regards to the emotional and broader societal impact of their beliefs.

## Methods

All experiments were conducted at the Connecticut Mental Health Center in strict accordance with Yale University’s Human Investigation Committee who provided ethical review and exemption approval (HIC# 2000026290). Informed consent was provided by all research participants.

### Experiment

A total of N = 1538 online participants were recruited through CloudResearch^46^. Two studies were conducted; a study to investigate the impact of an individual’s social network on their beliefs (*social network study*) and a study to replicate the relationship between task behavior and paranoia/conspiracy beliefs (*replication study*). All participants were compensated and bonused the same way as in the previous study^2^. See Supplementary Fig. 5 for demographic characterization of our data.

#### Social network study

A total of n = 372 participants of which 310 were individuals with low paranoia and 62 were individuals with high paranoia all completed the social version of the behavioral task. It is important to note that we originally had n = 405 participants who completed the social network survey; however, 33 individuals unsuccessfully removed duplicated names from their social network list on the survey (see p. 3–4 of *social_network_survey.pdf* located on our Github page), making it difficult to generate an accurate network description of their social circle. Additionally, of the 372 network participants, we only had data across anxiety, depression and paranoia—to calculate psychopathology (Fig. 3)—for n = 203 participants.

#### Replication study

A total of n = 1166 participants (plus n = 372 from the *social network study*) were combined, resulting in a count of 831 and 707 participants completing the nonsocial and social versions of the behavioral task, respectively, and in a count of 1206 and 332 individuals with low paranoia and high paranoia, respectively.

### Behavioral task

In this study, participants completed a three-option probabilistic reversal learning task (see previous studies^2,5,6^) in two different scenarios—one with a non-social aspect using a deck of cards and another with a social aspect using avatars representing partners. In the card deck scenario, participants were asked to choose from three decks with the goal of earning the most points. It was also mentioned that the deck with the highest reward could change. In the partner scenario, participants were asked to choose from three avatars and to imagine working on a group project with them. The avatars could represent helpful or hurtful partners and the partner that would give them the best chance to succeed on a project could also change. The contingencies of rewards initially followed a 90–50–10 pattern but switched to 80–40–20, making it more difficult to distinguish whether a loss was due to chance or a change in the best deck (or partner).

### Self-report questionnaires

After game completion, questionnaires were administered via Qualtrics. We collected demographics (age, sex, race, educational attainment, income, and political affiliation; see Supplementary Fig. 5), self-report psychopathology—paranoia, anxiety and depression—and conspiracy beliefs—general, coronavirus vaccine, and QAnon. For paranoia, we used the revised Green et al., Paranoid Thoughts Scale (R-GPTS)—subdivided into two subscales to assess ideas of reference (part A) and ideas of persecution (part B)—to measure self-reported levels of paranoia^26^ with a clinical cut-off of 11 (i.e., ‘high’ implies scores $\ge 11$ on part B of the R-GPTS). For anxiety (we used the Beck Anxiety Inventory (BAI^47^), and, for depression, we used the Beck Depression Inventory II (BDI-II^48^). For the conspiracy beliefs, we looked at general conspiracies (*Gen-conspiracy*), covid19 vaccine-related (*C19-conspiracy*) and QAnon-related (*Q-conspiracy*). We used five questions as a measure of an individual’s beliefs in general conspiracies^49^.

1. Much of our lives are being controlled by plots hatched in secret places.
2. Even though we live in a democracy, a few people will always run things anyway.
3. The people who really ‘run’ the country are not known to the voters.
4. Big events like wars, the recent recession, and the outcomes of elections are controlled by small groups of people who are working in secret against the rest of us.
5. High-ranking Democratic Party officials and US restaurants are connected to a global child-trafficking ring.

We adopted a 5-point scale: strongly disagree (1), disagree (2), neither agree nor disagree (3), agree (4) and strongly agree (5). A higher score indicates greater endorsement of a theory.

To measure covid19 vaccine beliefs, we used the following five questions^50^.

1. The coronavirus vaccine will contain microchips to control people
2. Coronavirus was created to force everyone to get vaccinated
3. The vaccine will be used to carry out mass sterilization
4. The coronavirus is bait to scare the whole globe into accepting a vaccine that will introduce the ‘real’ deadly virus
5. The World Health Organization already has a vaccine and are withholding it

We adopted a 7-point scale: strongly disagree (1), disagree (2), somewhat disagree (3), neutral (4), somewhat agree (5), agree (6), strongly agree (7). A higher score indicates greater endorsement of a theory.

To measure QAnon conspiracy belief, we asked how strongly the associate with the “QAnon” movement from 0 (they absolutely do not) to 100 (they absolutely do)^49^.

### Social network survey

Upon completion of self-report questionnaires, participants recruited to the *social network study* were asked to complete a social network survey. We modified an online questionnaire^20^ to solicit an individual’s personal network, including the specific network members, their relationships and what members of their network believe—with a particular focus on conspiratorial beliefs. The questionnaire begins by querying names (first name or initials only) of people in the individual’s life based on three central questions: (1) who they have discussed important matters with, (2) socialized with, or (3) sought support in the last three months.

The number of people who could be named was not capped but, for clarity, we considered the maximum number of network edges to be only the connections between each pair of the first 15 persons in the network (i.e., the first five persons listed under each of those three questions).

We then queried the strength of the ego-alter relationship, where ‘ego’ is defined as the individual who is taking our task and survey, and ‘alter’ is defined to be other members who the ego mentioned to be in their social network.

The strength of the **ego-alter** relationship was defined as weak—if ‘not especially close’—and strong—if ‘especially close’.

The strength of **alter-alter** relationships was defined as either stranger, in-between (weak) or especially close (strong).

For generating the ego-alter relationships, we asked whether “{unique name 1}” is especially close or not especially close with the ego, and for generating the alter-alter relationships, we asked whether “{unique name 1}” is a total stranger, especially close, or in-between with the other members that the ego entered into the survey. On average respondents (i.e., ego) entered 5 unique social network members (i.e., alters), of whom, 70% were especially close and 30% were not especially close to the ego.

Finally, we ascertained what egos believed about alters’ beliefs in conspiracies (based on the five-item general conspiracy questionnaire; see p. 36–41 of *social_network_survey.pdf* located on our Github page).

### Social network measures

In this study, we focused on some of the basic network measures and characteristics used in many social network studies^20^. Each of these measures are defined below.

1. **Network Size**: network measure of the number of members; larger values indicate a bigger social circle.
2. **Strong Ties**: network measure of ‘especially close’ relationships between members; larger values indicate members are closely connected with each other.
3. **Density**: network measure of the ratio of the number of existing relationships and the number of potential relationships in a network; larger values indicate more members know each other.
4. **Centralization**: network measure of how centered a network is on a single node; larger values indicate more alters directly connected to ego.
5. **Constraint**: network measure of structural holes (i.e., gaps in connectedness amongst nodes); larger values indicate interconnectedness amongst members. A node (or individual) will have the highest constraint if the node is connected to just a few other nodes and all the nodes are connected with each other.
6. **Kinship**: network characteristic of relatedness amongst members; larger values indicate higher proportion of family members (e.g., parents, siblings, spouse) than non-family members (e.g., friend, advisor, co-worker) in the network.

### Social network assumed shared belief

A key social network measure in our study is what we coin *assumed shared* belief. That is, *do egos believe their alters share their conspiracy beliefs?* We formulated a metric of *assumed shared belief* of individual $i$ (${\ddot{B}}_{i}$). It uses the ego’s responses to the above five general conspiracy questions (${{\varvec{e}}}_{i}$; a vector of mean responses duplicated in vector length to the number of alters in the ego’s network) and what the ego assumes each alter believes about the same conspiracy theories (${{\varvec{a}}}_{i}$; a vector of mean responses from each alter).

We calculate *assumed shared belief* of individual $i$ for all individuals $N$ as the weighted sum similarity

$$\ddot{{B}_{i}}= \sum_{i}^{{A}_{i}}{{\varvec{s}}}_{i}*{{\varvec{w}}}_{i}, \, for \, i=1, 2 , 3,\dots ,N$$

where ${A}_{i}$ represents the number of alters in ego’s $i$ network and ${{\varvec{s}}}_{i}={a}_{i}/{e}_{i}$ is a vector of similarity scores of the responses between the egos and their alters, weighted by a vector of network tie strength weights ${{\varvec{w}}}_{i}=({w}_{1},{w}_{2},\dots ,{w}_{{A}_{i}})$ where ${w}_{i}\in (\text{0,1})$. Larger values of $\ddot{{B}_{i}}$ indicate greater *assumed shared belief*.

### Behavioral analysis

Based on our prior work with the task in this population^2,5^, we analyzed tendencies to choose alternative decks after positive feedback (win-switch) and select the same deck after negative feedback (lose-stay). Win-switch rates were calculated as the number of trials in which the participant switched after positive feedback divided by the number of trials in which they received positive feedback. Lose-stay rates were calculated as number of trials in which a participant persisted after negative feedback divided by total negative feedback trials.

### Cluster analysis

K-means clustering was used to identify key social network profiles of individuals that differentiate belief-updating behavior. To determine the optimal number of clusters, k, we employed the elbow method (See Supplementary Fig. 4A). This method iteratively performs k-means for different k values and plots against a within-cluster sum of squares (WCSS) metric to select the optimal number of clusters. A bend in the plot indicates better separability of the data points. We then computed the principal components using PCA to visualize the clusters of the social network profiles.

### Multiple regression analysis

We conducted two multiple linear regression analyses. The first (Fig. 3A) attempted to predict psychopathology (a composite score of anxiety, depression and paranoia) from four network features—network size (SIZE), total number of strong ties (TIES), kinship (KIN) and assumed shared belief (BELIEF)—which were chosen since these features were noticeably different in cluster 3 from all other clusters. We fit a 15-predictor psychopathology model and performed backward stepwise regression to find the model that best explains our data (See Supplementary Table 1).

### Reduced model

$$\widehat{y} = {{\varvec{\beta}}}_{0} +{{\varvec{\beta}}}_{1}*{\text{X}}_{\text{SIZE}}+{{\varvec{\beta}}}_{2}*{\text{X}}_{\text{TIES}}+{{\varvec{\beta}}}_{3}*{\text{X}}_{\text{KIN}}+{{\varvec{\beta}}}_{4}*{\text{X}}_{\text{BELIEF}}+{{\varvec{\beta}}}_{5}*{\text{X}}_{\text{SIZE}*\text{TIES}}+{{\varvec{\beta}}}_{6}*{\text{X}}_{\text{TIES}*\text{KIN}}+{{\varvec{\beta}}}_{7}*{\text{X}}_{\text{SIZE}*\text{BELIEF}}$$

The second (Fig. 3B) attempted to predict initial prior volatility (${\mu }_{3}^{0}$) from paranoia and the four network features mentioned above. We fit a 31-predictor full model and, similarly, performed backward stepwise regression to obtain a 13-predictor reduced model (See Supplementary Table 2).

### Reduced model

$$\widehat{y} = {{\varvec{\beta}}}_{0} +{{\varvec{\beta}}}_{1}*{\text{X}}_{\text{PARANOIA}}+{{\varvec{\beta}}}_{2}*{\text{X}}_{\text{SIZE}}+{{\varvec{\beta}}}_{3}*{\text{X}}_{\text{TIES}}+{{\varvec{\beta}}}_{4}*{\text{X}}_{\text{KIN}}+{{\varvec{\beta}}}_{5}*{\text{X}}_{\text{BELIEF}}+{{\varvec{\beta}}}_{6}*{\text{X}}_{\text{PARANOIA}*\text{SIZE}}+{{\varvec{\beta}}}_{7}*{\text{X}}_{\text{PARANOIA}*\text{TIES}}+{{\varvec{\beta}}}_{8}*{\text{X}}_{\text{PARANOIA}*\text{KIN}}+{{\varvec{\beta}}}_{9}*{\text{X}}_{\text{PARANOIA}*\text{BELIEF}}+{{\varvec{\beta}}}_{10}*{\text{X}}_{\text{TIES}*\text{BELIEF}}+{{\varvec{\beta}}}_{11}*{\text{X}}_{\text{KIN}*\text{BELIEF}}+{{\varvec{\beta}}}_{12}*{\text{X}}_{\text{PARANOIA}*\text{TIES}*\text{BELIEF}}+{{\varvec{\beta}}}_{13}*{\text{X}}_{\text{PARANOIA}*\text{KIN}*\text{BELIEF}}$$

### Computational modeling

The Hierarchical Gaussian Filter (HGF) toolbox v5.3.1 is freely available for download in the TAPAS package at https://translationalneuromodeling.github.io/tapas^51–53^. We installed and ran the package in MATLAB and Statistics Toolbox Release 2016a (MathWorks®, Natick, MA).

We estimated perceptual parameters individually for the first and second halves of the task (i.e., for trials 1–80 and then trials 81–160). Each participant’s choices (i.e., deck 1, 2, or 3) and outcomes (win or loss) were entered as separate column vectors with rows corresponding to trials. Wins were encoded as ‘1’, losses as ‘0’, and choices as ‘1’, ‘2’, or ‘3’. We selected the autoregressive 3-level HGF multi-arm bandit configuration for our perceptual model and paired it with the softmax-mu03 decision model.

### Statistics

Statistical analyses and effect size calculations were performed with an alpha of 0.05 and two-tailed p-values in RStudio: Integrated Development Environment for R, Version 1.3.959. For the regression analysis, we used the F-statistic to test the significance of regression coefficients. To account for the non-normal distributions between paranoia groups, we used non-parametric tests for group comparisons (Unpaired two-samples Wilcoxon Test) and correlations (Spearman, ρ).

HGF parameter estimates and behavioral patterns (win-switch and lose-stay rates) were analyzed with unpaired two-samples Wilcoxon Test between paranoia groups.

### Parameter recovery on simulated data

We fit our HGF model to our observed PRL task data (true), estimating belief model parameters. We demonstrate parameter recovery by attempting to recover the parameter estimates—originally estimated from the true choice data—on simulated choice data. We simulated individual choice data using *tapas_simModel()* where the input parameters included the true response data (choices/outcomes), the perceptual model, the true model parameter estimates, and the decision model. This was performed for i = 10 iterations, generating 10 different sets of simulate choice data for each individual. We re-fit the HGF, running *tapas_fitModel(),* but now using the simulated choice data to generate a set of belief parameters for every subject on each iteration. The simulated choices recapitulate the win-switch behavioral effect (Supplementary Fig. 3A [top]) and the prior on volatility (Supplementary Fig. 3A [bottom]).

## Supplementary Information

Supplementary Information.

## Data Availability

The data that support this paper are available at https://github.com/psuthaharan/social-network-prl.

## Code availability

The code used to analyze the data and generate the figures is available at https://github.com/psuthaharan/social-network-prl.
