# Supplementary material for: Assumed shared belief about conspiracy theories in social networks protects paranoid individuals against distress
Source: Sci Rep. 2023 Apr 13;13:6084. doi: 10.1038/s41598-023-33305-w (PMC10100615; doi:10.1038/s41598-023-33305-w)
Supplement: Supplementary file 1 — Supplementary Information. [file 41598_2023_33305_MOESM1_ESM.docx]

Supplementary Materials

**Assumed shared belief about conspiracy theories in social networks protects paranoid individuals against distress**

Praveen Suthaharan^1,2,3^ and Philip R. Corlett^2,3,4,5*^

^1^Interdepartmental Neuroscience Program, Yale University, New Haven, CT, USA

^2^Kavli Institute for Neuroscience, Yale University, New Haven, CT, USA

^3^Department of Psychiatry, Connecticut Mental Health Center, Yale University, New Haven, CT, USA

^4^Department of Psychology, Yale University, New Haven, CT, USA

^5^Wu Tsai Institute, Yale University, New Haven, CT, USA

*Corresponding Author email: [philip.corlett@yale.edu](mailto:philip.corlett@yale.edu)


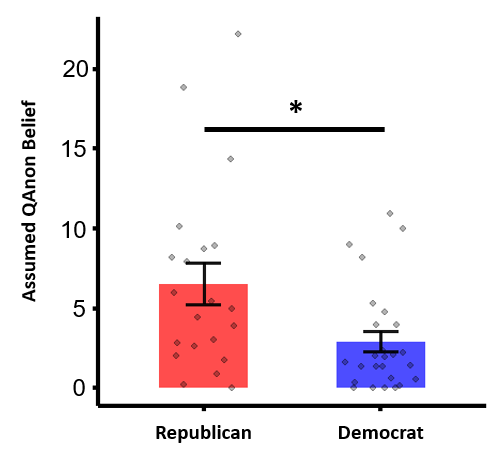


**Supplementary Figure 1. A sacred canopy for political conspiracies.** Republicans assume members of their social network share their QAnon beliefs more so than Democrats ($t_{30}=2.5, p=0.02$).


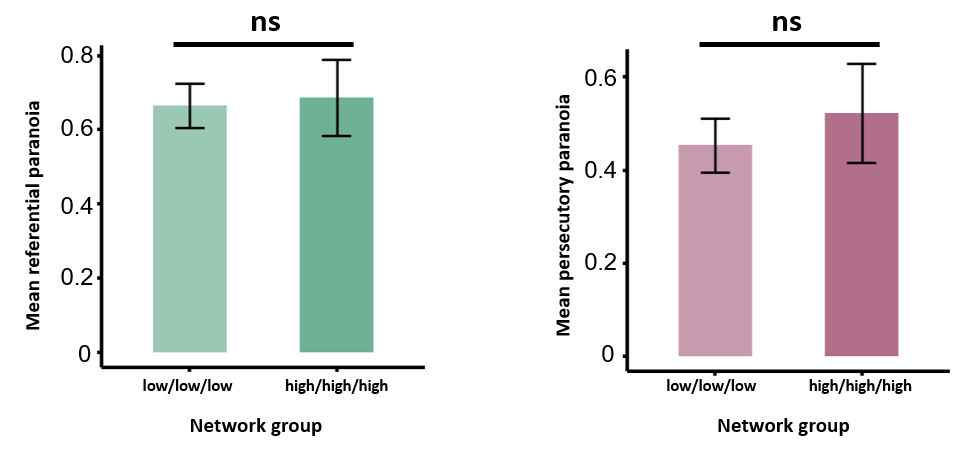


**Supplementary Figure 2. Referential and persecutory belief differences between network groups.** We grouped individuals into those with larger network size/greater strong ties/higher assumed belief) and those who do not. There was no elevation of self-referentiality in the people with low assumed belief, weak ties, and low assumed shared belief (Moderate evidence for null hypothesis of no difference between referential paranoia: $BF_{10}=0.149;persecutory paranoia: BF_{10}=0.175$).

This was a follow up test, aimed at exploring the relative contributions of referential paranoia (hints, events, stimuli, have personal significance) versus persecutory paranoia (people have ill intent towards me) – both measured with the R-GPTS. Our intention with the test was to scrutinize a theory of paranoia and conspiricism that ventures ego centraility as particularly relevant to pathological paranoia – put simply, something is more like a delusion if the person endorsing it believes they are at the center of the plot. We did not find any evidence in favor of this hypothesis.

**
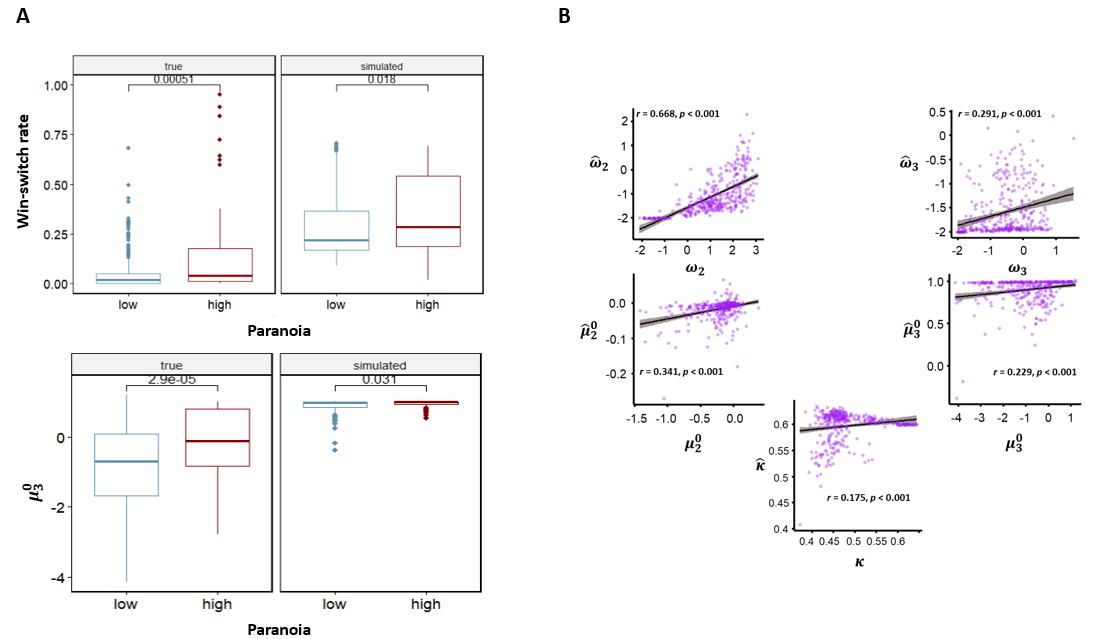
**

**Supplementary Figure 3. Parameter recovery of task behavior and beliefs from our social network participant pool.** We illustrate parameter recovery performance on simulated choice data from the collected choice data and estimated model parameters in our social network participant pool. (A) *Recovered paranoia group differences.* We estimated parameters from choice data simulated from the participants' parameters.  The simulated choices recapitulate the win-switch behavioral effect and the prior on volatility - simulated paranoid agents win-switch more and have stronger priors on volatility. (B) *Comparing estimated and simulated belief parameters.* We observe significant but moderate correlation between the true parameters (estimated from collected data) and recovered parameters (estimated from simulated data; denoted by the ‘hat’ symbol). See *Methods* for details regarding parameter recovery. Recapitulating the observed group differences in simulated data represents a critical demonstration that we have the correct model.

See: **The Importance of Falsification in Computational Cognitive Modeling**

Stefano Palminteri, Valentin Wyart, Etienne Koechlin

Trends Cogn Sci. 2017 Jun;21(6):425-433.

doi: 10.1016/j.tics.2017.03.011. Epub 2017 May 2.


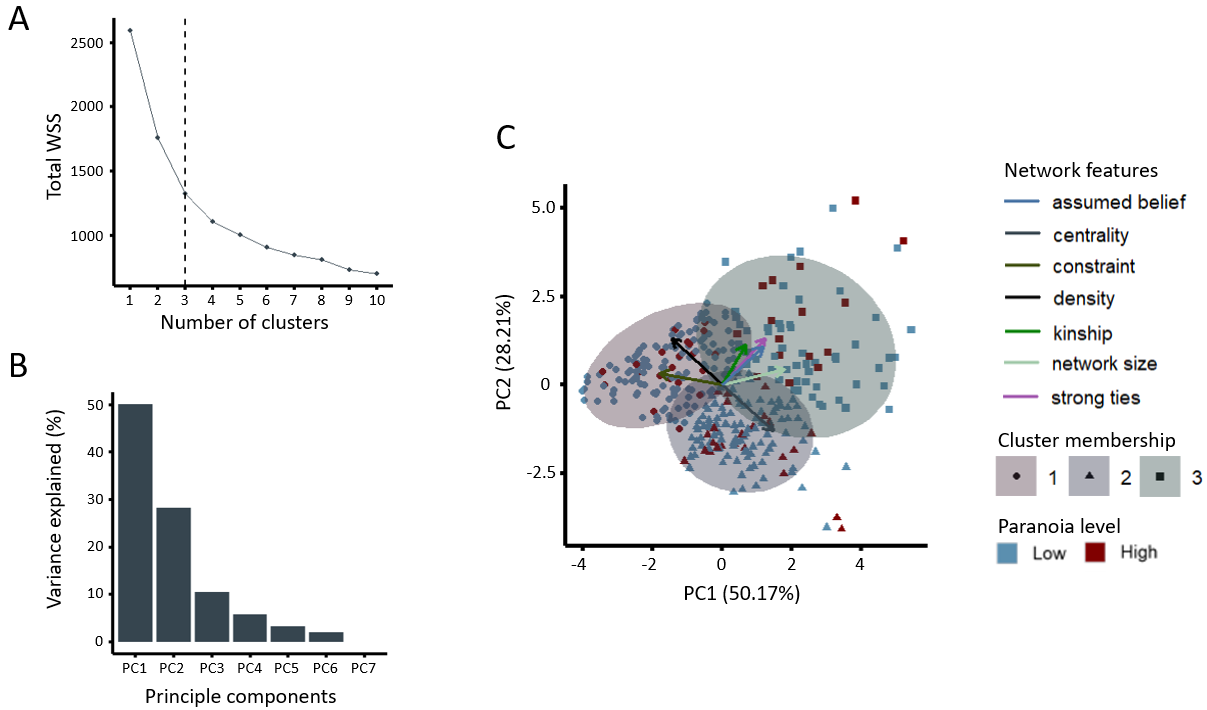


**Supplementary Figure 4. Dimensionality reduction by k-means clustering and principal component analysis (PCA).** We cluster our participants (N=372) – characterized on seven social network features – using k-means clustering, and visualize the cluster membership using principal component analysis (PCA). (A) *Elbow method for choosing number of clusters.* Our elbow test reveals k = 3 to 5 clusters may be sufficient to capture separability within data points (as measured by WSS). On visual inspection, we decided to choose k=3 to maintain a decent sample size in each cluster group for sufficient statistical analyses. (B) *Scree plot for visualizing variance explained.* A scree plot can be used to diagnose the quality of the data; our plot shows a steep drop-off in explained variance after the first few components, leveling off into a more gradual decline. However, if we saw a steep decline with no clear point of inflection this could suggest a high degree of collinearity in our data. Given we do not see that, we can say that our seven network features contribute new information to the explanation of variability. Moreover, it is important to recognize that, together, the first two principal components explain a large percentage (~ 70%) of the variability in our data. (C) *K-means clustering via PCA.* We illustrate clustering of our network data using k=3 clusters (based on the elbow test) in a dimensionally-reduced (i.e., dim = 7 to dim = 2) space represented by PC1 and PC2 which, together, explain about 72% of the variance in our data.


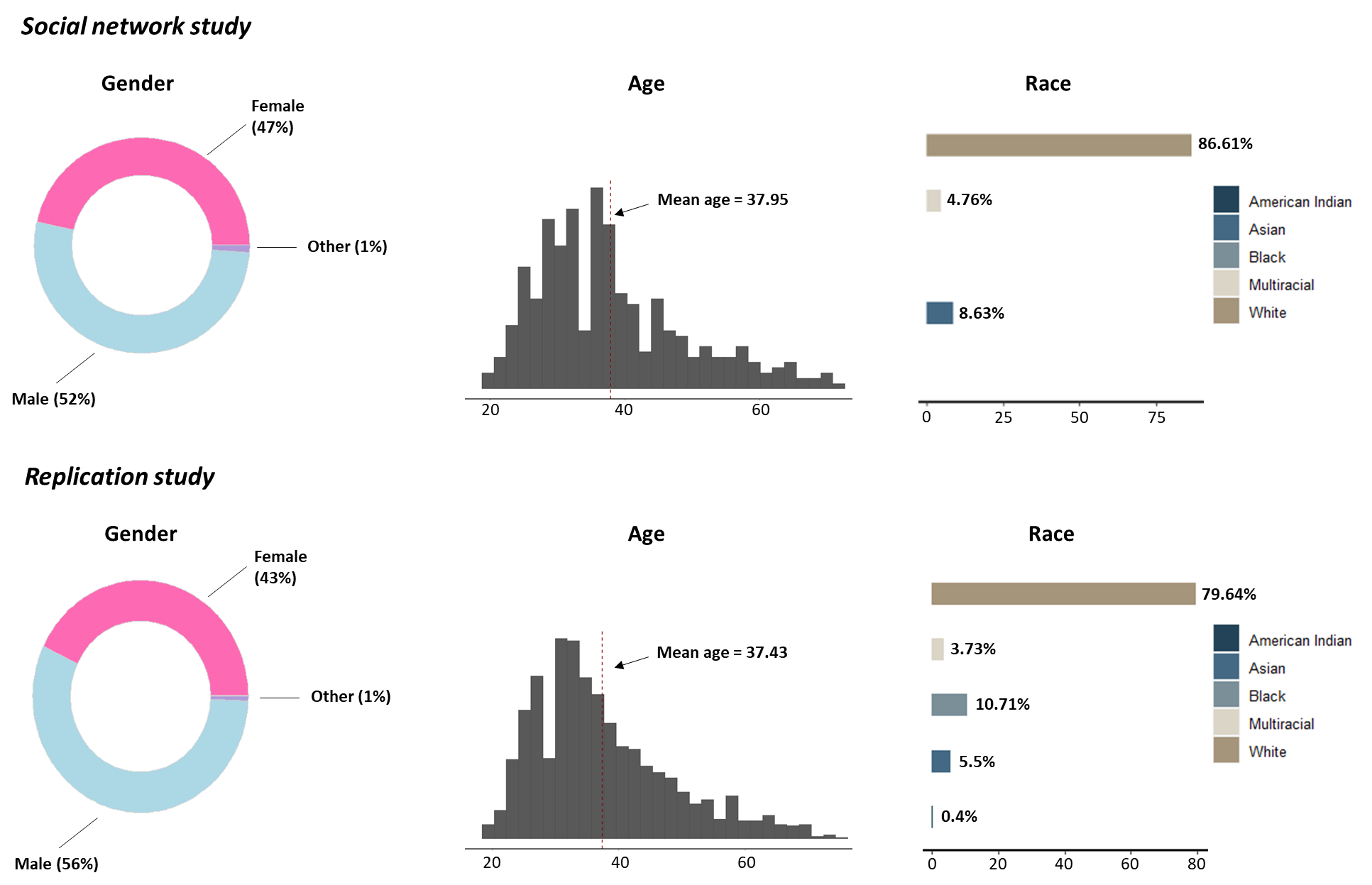


**Supplementary Figure 5. Demographics by study.** We characterize our participant data by gender, age and race demographics.

**Supplementary Table 1.** Regression to predict psychopathology

| Predictors | **Full model** | **Reduced model** |
| --- | --- | --- |
|  |  |  |
| **SIZE**  **TIES**  **KIN**  **BELIEF**  **SIZE*TIES**  **SIZE*KIN**  **TIES*KIN**  **SIZE*BELIEF**  **TIES*BELIEF**  **KIN*BELIEF**  **SIZE*TIES*KIN**  **SIZE*****TIES*BELIEF**  **SIZE*KIN*BELIEF**  **TIES*KIN*BELIEF**  **SIZE*TIES*KIN*BELIEF**  **Adjusted R^2^** | -0.0076  -0.2726  0.2331  0.8087  0.0403  -0.0370  0.0594  -0.2846  0.1893  0.1703  -0.0064  -0.0201  0.0182  -0.0839  0.0065  0.08 | -0.0377 **  -0.0476  0.2949 *  1.1350  0.0175 **  -  -0.0238 **  -0.2900 *  -  -  -  -  -  -  -  0.10 |
| ***p*-value** | 0.008 | < 0.001 |
|  |  |  |

*p ≤ 0.05, **p ≤ 0.01, ***p ≤ 0.001; SIZE = Network Size, TIES = Strong Ties,

KIN = Kinship, BELIEF = Assumed shared belief; PARANOIA = Self-report GPTS-B

**Supplementary Table 2.** Regression to predict initial prior volatility

| Predictors | **Full model** | **Reduced model** |
| --- | --- | --- |
|  |  |  |
| **PARANOIA**  **SIZE**  **TIES**  **KIN**  **BELIEF**  **PARANOIA*SIZE**  **PARANOIA*TIES**  **SIZE*TIES**  **PARANOIA*KIN**  **SIZE*KIN**  **TIES*KIN**  **PARANOIA*BELIEF**  **SIZE*BELIEF**  **TIES*BELIEF**  **KIN*BELIEF**  **PARANOIA*SIZE*TIES**  **PARANOIA*SIZE*KIN**  **PARANOIA*TIES*KIN**  **SIZE*TIES*KIN**  **PARANOIA*SIZE*BELIEF**  **PARANOIA*TIES*BELIEF**  **SIZE*TIES*BELIEF**  **PARANOIA*KIN*BELIEF**  **SIZE*KIN*BELIEF**  **TIES*KIN*BELIEF**  **PARANOIA*SIZE*TIES*KIN**  **PARANOIA*SIZE*TIES*BELIEF**  **PARANOIA*SIZE*KIN*BELIEF**  **PARANOIA*TIES*KIN*BELIEF**  **SIZE*TIES*KIN*BELIEF**  **PARANOIA*SIZE*TIES*KIN*BELIEF**  **Adjusted R^2^** | -0.6989  -0.4123 *  -0.0055  -0.5174  -1.4413  0.3169  -0.1154  0.0496  0.3752  0.0786  -0.0035  3.5145  0.2661  0.0954  0.5441  -0.0172  -0.0581  0.0464  -0.0065  -0.5717  -0.0606  -0.0419  -1.1598  -0.0956  0.0081  -0.0009  0.0310  0.1936  -0.0545  0.0053  -0.0008  0.11 | 0.0028  -0.1343 *  0.1951 ***  -0.3096 **  -0.0420  0.1312 *  -0.1439 ***  -  0.2754 **  -  -  -0.0353  -  -0.0700  0.1230  -  -  -  -  -  0.0899 *  -  -0.3007 **.**  -  -  -  -  -  -  -  -  0.16 |
| ***p*-value** | 0.011 | < 0.001 |
|  |  |  |

*p ≤ 0.05, **p ≤ 0.01, ***p ≤ 0.001; SIZE = Network Size, TIES = Strong Ties,

KIN = Kinship, BELIEF = Assumed shared belief; PARANOIA = Self-report GPTS-B
